# Supplementary material for: Resilient SARS-CoV-2 diagnostics workflows including viral heat inactivation
Source: PLoS One. 2021 Sep 15;16(9):e0256813. doi: 10.1371/journal.pone.0256813 (PMC8443028; doi:10.1371/journal.pone.0256813)
Supplement: Attachment — Submitted filename: responses.odt [file pone.0256813.s002.odt]

School of Immunology and Microbial Sciences
King’s College London

Asthma UK Centre in Allergic Mechanisms of Asthma

Dr Rocio T Martinez-Nunez
Dept. Infectious Diseases
5th Floor Tower Wing | Guy’s Campus
King’s College London
London SE1 9RT
Tel: +44(0)2071880595 
rocio.martinez_nunez@kcl.ac.uk


April 16th 2021,
Dear Editorial Board of PLOS One,


Point by Point answers
Reviewer #1: 
General comments:
1) Please put your work in context of other similar work, both in the introduction and the discussion. There are currently no such references in the introduction, while there is e.g.:
- Merindol N, Pépin G, Marchand C, et al. SARS-CoV-2 detection by direct rRT-PCR without RNA extraction. J Clin Virol. 2020;128:104423. doi:10.1016/j.jcv.2020.104423
- Fomsgaard AS, Rosenstierne MW. An alternative workflow for molecular detection of SARS-CoV-2 - escape from the NA extraction kit-shortage, Copenhagen, Denmark, March 2020. Euro Surveill. 2020;25(14):2000398. doi:10.2807/1560-7917.ES.2020.25.14.2000398
- Mancini F, Barbanti F, Scaturro M, et al. Laboratory management for SARS-CoV-2 detection: a user-friendly combination of the heat treatment approach and rt-Real-time PCR testing. Emerg Microbes Infect. 2020;9(1):1393-1396. doi:10.1080/22221751.2020.1775500
We agree and have now included a more updated bibliography in our manuscript thank you.

2) No statistical analysis is performed to determine whether significantly better or worse results are obtained using the different methods. This is necessary to be able to draw conclusions. In particular, in the Discussion p 6: FastVirus and Luna showing better results but PCRBio also being also a good alternative.
We fully agree with the Reviewer and thank them for this input. We have provided statistical analyses in our graphs and text supporting our observations. ANOVA analysis showed no significant difference overall although multiple testing (with p-value correction) showed that Luna provided slightly lower Ct values as compared with FastVirus. One sample was positive and detected by both FastVirus and Luna and missed by PCRBio.

3) It should be clearly stated in the discussion that only in the event of shortages should heat inactivation be employed, subject to further validation. This manuscript does not in itself provide any evidence of superiority of heat activation over standard workflows. For the same reason it should not be stated that N1 and N2 be preferred vs RdRP - the N targets are actually often less sensitive within kits that employ several targets.
We have revised our statistics and now provide ~90 samples comparing heat vs non-heat inactivation at a higher temperature of 90°C which appears to decrease sensitivity and thus we propose the use of 70°C instead. Importantly we provide plaque assays for both the original and B.1.1.7 variants. We also cite other work where heat inactivation is implemented. We also cite the work by Vogels et al. 2020 (Nature Microbiology) which shows decreased sensitivity of RdRP detection, possibly due to the presence of an increased number of subgenomic RNAs for the N gene as per the work of Kim et al., 2020 (Cell).

Specific comments:

1) References to preprints (reference 5) should be avoided.

We have revised this thank you.

2) Page 5, end. It should be clarified whether negative donors are PCR- symptomatic patients, or rather they healthy individuals and/or pre-outbreak (before 2020) samples. The latter is much preferred to avoid false negative samples with virtual certainty.
Thank you for this suggestion. Negative samples were considered as those diagnosed as negative during the pandemic, we do not have swab material pre-pandemic. This is now clarified in the text.

3) Fig 2A should have same inverse X-axis, same as Fig 3 (or vice versa), for consistency.
We have tried plotting this as such but considered that since we are plotting copy numbers vs swab dilutions in these figures, and the R2 is clarified, we could leave this panel as it was. 

4) Fig 4D. A Ct of 40 realistic does not seem realistic to evaluate results, if about half of the negative samples (regardless of being heat inactivated or not) fall below that.
We are unsure about how to interpret this comment from Reviewer one. A Ct of 40 is considered by some laboratories as a positive sample, since it depends on the evaluation of clinical history and manifestations. We have left our plots to reflect our data. 
Reviewer#2: 
This study evaluates kit-free protocols for SARS-Cov-2 detection and provides a recommended protocol for heat inactivation that would allow testing to be completed at a lower biosafety level from the current BSL/CL-3 requirement. The findings are highly relevant and timely to enable decentralized commercial and academic laboratories to participate in COVID testing in order to meet immediate needs for greater testing capacity. Strengths of this work include: (i) the selection of multiple, popular RNA extraction methods with comparison to the gold standard QIAGEN; (ii) evaluation of several commercially available RT-PCR mixes and both primer sets recommended by CDC/PHE; (iii) establishing a heating protocol that does not impact PCR results but would allow non-BSL/CL-3 containment. The methods, results, and conclusions are clear and compelling. The only weakness that I see is that there are no standard error/deviation bars on any of the graphs even when the samples were described as having been run with replicates.
Thank you for the kind comments of Reviewer 2. We have now performed statistics in our graphs, further explained replicates and provided an increased number of validations.

Reviewer#3: 
- The current work has two laudable goals: 1) determine the relative performance of different commercial RNA extraction methods, combined with different commercial master mixes, for the RT-qPCR detection of SARS-CoV-2 in patient samples; 2) determine if heat treatment has any effect on assay sensitivity. The first goal can address supply chain restrictions that limit our testing capacity, and the second goal would address biosafety concerns in sample processing, thus helping to expand testing centers. The overarching conclusions of the authors is that different extraction methods and master mixes perform comparably, and that heat treatment does not significantly impact test sensitivity.
None of the results are analyzed statistically, and we rely on subjective assessment of results. None of the author’s conclusions (summarized here) are adequately supported:
• Heat has minimal impact on sensitivity.
• All extraction methods yield similar results.
• FastVirus and Luna were more sensitive.
• N detection was more sensitive that detection of RdRp, particulary

Thank you for these points. We have now provided statistical analyses of all our observations and increased the sample number for heat inactivation using 90°C. We believe that the new version of our work supports the statements above. We observe decreased sensitivity when heating samples at 90°C but not when heating at 70°C, discuss the extraction methods based on statistics and analysis of individual patients, as this Reviewer helped us to do. We provide statistics showing better performance of Luna. We also provide citations to work observing less sensitivity of RdRP (Vogels et al. 2020, Nature Microbiology), possibly because N renders more subgenomic RNAs (Kim et al., 2020, Cell).
- Heat treatment: Data in figure 4A-C convincingly show that heat treatments known to inactivate virus does not appear to influence results of RT-qPCR using patient samples. Note that the authors indicate that the samples analyzed in figure 4A are the same as those analyzed in figure 3, although this is true of only two of the five patients analyzed in figure 4A (CPS_11 and CPS_21), and there is no overlap with samples analyzed in 4B, 4C or 4D. The Ct for from patient CPS_21 is approximately 25 when the sample is heat treated (figure 4A; sample analyzed using the FastVirus master mix) and yet the value is <15 in figure 3 (not heat treated, Luna master mix). The difference in Ct reflects orders of magnitude difference in the presumed target copy number (based upon data shown in figure 1). This raises concern that heat treatment might affect sensitivity, perhaps dependent upon the master mix employed in the RT-qPCR.
Upon this helpful observation by Reviewer 3, we have revised our data. We found that Figure 4A was not informative without the side-by-side comparison of no heat inactivation of these samples, which has to be done at the same time in the same qPCR and cannot be done comparing different plates/days. Since we did not have the samples to evaluate this any longer we have decided to remove that panel and provide a new dataset to support our observations of heat inactivation by comparing a higher temperature with a shorter treatment (90°C at 10 mins, now Figure 5D). As the Reviewer suggested, high heat decreases sensitivity although this does not seem to be the case with treatment of 70°C, which we have now evaluated and provide statistical evidence for.

- Extraction method: Figure 3 would seem to indicate that RNA extraction method is not a significant variable and thus should perform well in samples with low viral content, based upon analysis of serially diluted patient samples and use of the Luna master mix with N1 and N2 primers. However, in figure 2B, RNA extraction method appears to have a significant impact on sensitivity in patient CPS_84. The Beckman extraction method yields a product at approximately 20 cycles, whereas an additional ten cycles are required using the QIAamp and Omega kits together with the FastVirus master mix. Using the Omega extraction kit and the Luna master mix (figure 2C), with see increased Ct’s for three of the four patient samples. And the standard curves (figure 3B) are not acceptable for two patients for which the extraction was performed using the Omega kit and the N2 primer. The samples are from CPS_21 and CPS_79; an R2 of 0.99 was only obtained after ignoring results from the 10-1 dilution. This raises concerns regarding the choice of primer pairs in combination with extraction method and master mix – a concern already raised with primer recognizing the RdRp coding region. In patient sample CPS_11 (figure 3B), we also see an additional five cycles required to achieve a positive result for both N1 and N2 primer pairs relative to sample extractions using.
This is an excellent point by this Reviewer and we sincerely agree. We have now revised our text including these observations, which support a superior performance from Beckman extraction over the other methods based on analysing individual patient’s data – despite our ANOVA data showing similar performance when all are considered together. 

- Master Mix: The authors conclude that FastVirus and Luna master mixes yield more sensitive detection than PCRBiosystems, but this reviewer cannot identify data supporting this conclusion.
Thank you, we have now included statistical analysis that supports our statements, and Luna appears to yield lower Ct values as compared with FastVirus. PCRBio missed one positive sample captured with the other two MasterMixes, although statistical analysis showed no difference between all three methods when compared together.
- Sensitivity: A limitation of the study reflects the fact that none of these data can be related to viral genome copy number. The authors acknowledge this fact, lacking either in vitro transcribed RNA or source of cultured virus to use as standards. Using plasmid as a standard does show that they can detect 1 copy of cloned nucleocapsid sequence at a Ct less than 40 when using the N1 primer (but not the N2 primer). Sensitivity is likely less for actual virus when factoring in the efficiency of the RT step. Limitations in sensitivity likely contributes to the extreme variability of results shown in figure 4D. Samples once testing negative will variably yield positive results. The majority of samples upon which this study is based (comparing impact of RNA extraction method or RT-qPCR master mix) occurs in patient samples with higher viral loads reflected in Ct’s ≤ 30. In this range, there may be lesser impacts of using different methods.
Thank you for these observations and comments. Our limit of detection experiments in Figure 3 were done with diluted swab material which we believe better reflect limit of detection than with plasmid. We have acknowledged the limitation of our study in the text and now provide another new limit of detection for the new B.1.1.7 variant based on cultured virus and pfu/mL in Figure 4.

If anything, results would seem to reinforce the importance of assay validation for any combination of extraction method, master mix and primers employed, and any pretreatment of samples.
We fully agree with this helpful comment and have now highlighted this as part of essential work to be carried out when validating new methods of detection of SARS-CoV-2, as well as the need for using clinical samples.
